# Supplementary material for: Biophysical and X-ray structural studies of the (GGGTT)3GGG G-quadruplex in complex with N-methyl mesoporphyrin IX
Source: PLoS One. 2020 Nov 18;15(11):e0241513. doi: 10.1371/journal.pone.0241513 (PMC7673559; doi:10.1371/journal.pone.0241513)
Supplement: S1 Table — (DOCX) [file pone.0241513.s001.docx]

**S1 Table.** Occurrences of the T1 sequence in the human genome (GRCh38.p12 primary assembly) identified via BLAST.

| **Chromosome** | **Start position** | **End position** | **Significance** |
| --- | --- | --- | --- |
| 6 | 87229964 | 87229981 | Within zinc finger protein 292 gene |
| 7 | 132317250 | 132317267 | Complement of plexin A4 gene |
| 12 | 9924441 | 9924458 | Complement of C-type lectin domain family 2 member A gene |
|  | 9924446 | 9924463 | Complement of C-type lectin domain family 2 member A gene |
|  | 9924451 | 9924468 | Complement of C-type lectin domain family 2 member A gene |
|  | 68918645 | 68918662 | Complement of carboxypeptidase M gene |
| 14 | 99067501 | 99067518 | Complement of LOC107984696 ncRNA |
|  | 99067506 | 99067523 | Complement of LOC107984696 ncRNA |
|  | 99067532 | 99067549 | Complement of LOC107984696 ncRNA |
| 17 | 75933313 | 75933330 | Complement of fas binding factor 1 gene |
| 21 | 41156399 | 41156416 | ––– |
