# Supplementary material for: Biophysical and X-ray structural studies of the (GGGTT)3GGG G-quadruplex in complex with N-methyl mesoporphyrin IX
Source: PLoS One. 2020 Nov 18;15(11):e0241513. doi: 10.1371/journal.pone.0241513 (PMC7673559; doi:10.1371/journal.pone.0241513)
Supplement: S2 Table — (DOCX) [file pone.0241513.s002.docx]

**S2 Table.** Thermodynamic stability of T1, T7, and T8 in the presence of 2 eq. of NMM in 5K buffer.

|  | T_m_ *,* °C | ΔT_m_ relative to DNA alone *,* °C | ΔH, kcal/mol |
| --- | --- | --- | --- |
| T1 + NMM | 78 ± 1 | 20. ± 1 | 57 ± 2 |
| T7 + NMM | 71.9 ± 0.3 | 19.9 ± 0.4 | 53.0 ± 0.9 |
| T8 + NMM | 73.4 ± 0.4 | 17.0 ± 0.5 | 54.5 ± 0.6 |
