# Supplementary material for: Biophysical and X-ray structural studies of the (GGGTT)3GGG G-quadruplex in complex with N-methyl mesoporphyrin IX
Source: PLoS One. 2020 Nov 18;15(11):e0241513. doi: 10.1371/journal.pone.0241513 (PMC7673559; doi:10.1371/journal.pone.0241513)
Supplement: S4 Table — (DOCX) [file pone.0241513.s004.docx]

**S4 Table.** Groove widths in the T1-NMM structure (Å).

|  | **G-quartets** | **Groove 1** | **Groove 2** | **Groove 3** | **Groove 4** |
| --- | --- | --- | --- | --- | --- |
| Chain A | **3'** | 15.9 | 16.6 | --- | --- |
|  | **Middle** | 16.6 | 16.3 | 16.7 | 16.5 |
|  | **5'** | 16.2 | 15.7 | 16.3 | 16.1 |
| Chain B | **3'** | 15.9 | 16.9 | --- | --- |
|  | **Middle** | 16.3 | 16.5 | 16.9 | 16.9 |
|  | **5'** | 16.1 | 15.8 | 16.5 | 16.3 |
| **Average** | | **16.2 ± 0.3** | **16.3 ± 0.5** | **16.6 ± 0.3** | **16.5 ± 0.3** |

Based on the data above, the average groove widths for both chains for 3’ G-quartet, middle G-quartet, and 5’ G-quartet are 16.3 ± 0.5, 16.6 ± 0.2, and 16.1 ± 0.3 Å, respectively
