# Supplementary material for: Biophysical and X-ray structural studies of the (GGGTT)3GGG G-quadruplex in complex with N-methyl mesoporphyrin IX
Source: PLoS One. 2020 Nov 18;15(11):e0241513. doi: 10.1371/journal.pone.0241513 (PMC7673559; doi:10.1371/journal.pone.0241513)
Supplement: S5 Table — (DOCX) [file pone.0241513.s005.docx]

**S5 Table.** RMSD (Å) for the T1- and T7-NMM structures.

|  | DNA chain | | Overall | GQ core |
| --- | --- | --- | --- | --- |
| Within structure | T1_A | T1_B | 0.9 | 0.49 |
|  | T7_A | T7_B | 1.2 | 0.23 |
| Between structures | T1_A | T7_A | 2.0 | 0.64 |
|  | T1_A | T7_B | 1.1 | 0.61 |
|  | T1_B | T7_A | 1.2 | 0.66 |
|  | T1_B | T7_B | 1.0 | 0.66 |
|  | **Average** | | **1.3 ± 0.4** | **0.64 ± 0.02** |
