# Supplementary material for: Biophysical and X-ray structural studies of the (GGGTT)3GGG G-quadruplex in complex with N-methyl mesoporphyrin IX
Source: PLoS One. 2020 Nov 18;15(11):e0241513. doi: 10.1371/journal.pone.0241513 (PMC7673559; doi:10.1371/journal.pone.0241513)
Supplement: S6 Table — (DOCX) [file pone.0241513.s006.docx]

**S6 Table.** B-factors (Å^2^) for the T1- and T7-NMM structures.

|  | T1-NMM | T7-NMM |
| --- | --- | --- |
| Overall | 115.44 | 97.15 |
| GQ with loops | 115.36 | 97.75 |
| NMM | 104.77 | 94.01 |
| GQ core | 106.46 | 81.18 |
| TT loops | 148.13 | 128.85 |
| T overhangs | --- | 151.63 |
| All Ts | 148.13 | 131.87 |
