# Supplementary material for: Biophysical and X-ray structural studies of the (GGGTT)3GGG G-quadruplex in complex with N-methyl mesoporphyrin IX
Source: PLoS One. 2020 Nov 18;15(11):e0241513. doi: 10.1371/journal.pone.0241513 (PMC7673559; doi:10.1371/journal.pone.0241513)
Supplement: S7 Table — (DOCX) [file pone.0241513.s007.docx]

**S7 Table.** Distances (Å) between G-quartets; 3’G-quartets and NMM; and between GQ monomers in the T1- and T7-NMM structures.

|  | T1-NMM | | | T7-NMM | | |
| --- | --- | --- | --- | --- | --- | --- |
| Distance between | **Chain A** | **Chain B** | **Average** | **Chain A** | **Chain B** | **Average** |
| 5’- middle | 3.320 | 3.315 | **3.32** ± 0.01 | 3.44 | 3.39 | **3.41** ± 0.03 |
| Middle - 3’ | 3.40 | 3.31 | **3.36** ± 0.06 | 3.41 | 3.42 | **3.41** ± 0.01 |
| 3’ - NMM | 3.5 | 3.7 | **3.6** ± 0.2 | 3.62 | 3.65 | **3.64** ± 0.02 |
| 5’-5’ (between monomers) | --- | --- | **3.38** | --- | --- | **3.36** |
