# Supplementary material for: Biophysical and X-ray structural studies of the (GGGTT)3GGG G-quadruplex in complex with N-methyl mesoporphyrin IX
Source: PLoS One. 2020 Nov 18;15(11):e0241513. doi: 10.1371/journal.pone.0241513 (PMC7673559; doi:10.1371/journal.pone.0241513)
Supplement: S8 Table — (DOCX) [file pone.0241513.s008.docx]

**S8 Table**. Intramolecular helical twist (°) between each quartet pair in the T1- and T7-NMM structures.

|  | T1-NMM | | | T7-NMM | | |
| --- | --- | --- | --- | --- | --- | --- |
| Chain → | **A** | **B** | **Both** | **A** | **B** | **Both** |
| Between  5’ and middle quartets | 34 | 31 |  | 27.4 | 27 |  |
|  | 29 | 35 |  | 28.1 | 29 |  |
|  | 30 | 32 |  | 28.6 | 28 |  |
|  | 32 | 34 |  | 28.8 | 26 |  |
| Average | 31 ± 2 | 33 ± 2 | **32** ± 2 | 28.2 ± 0.6 | 27 ± 1 | **27.8** ± 0.9 |
| Between  middle and 3’ quartets | 27 | 24 |  | 27 | 28 |  |
|  | 29 | 24 |  | 28 | 26 |  |
|  | 27 | 25 |  | 28 | 27 |  |
|  | 28 | 27 |  | 31 | 29 |  |
| Average | 28 ± 1 | 25 ± 1 | **26** ± 2 | 29 ± 2 | 27 ± 2 | **28** ± 2 |
| Overall | **29** ± 4 | | | **28** ± 1 | | |
