# Supplementary material for: Biophysical and X-ray structural studies of the (GGGTT)3GGG G-quadruplex in complex with N-methyl mesoporphyrin IX
Source: PLoS One. 2020 Nov 18;15(11):e0241513. doi: 10.1371/journal.pone.0241513 (PMC7673559; doi:10.1371/journal.pone.0241513)
Supplement: S9 Table — A′ signifies a symmetry related chain A molecule. (DOCX) [file pone.0241513.s009.docx]

**S9 Table.** Intermolecular helical twist (°) at the dimer interface for T1-NMM and T7-NMM. A′ signifies a symmetry related chain A molecule.

| T1-NMM | | T7-NMM | |
| --- | --- | --- | --- |
| G pair | **Twist** | **G pair** | **Twist** |
| A1-A′16 | 117 | **A2-B17** | 115 |
| A6-A′11 | 115 | **A7-B12** | 118 |
| A11-A′6 | 115 | **A12-B7** | 118 |
| A16-A′1 | 117 | **A17-B2** | 118 |
| Average | **116** ± 1 | **Average** | **117** ± 2 |
