# Supplementary material for: Biophysical and X-ray structural studies of the (GGGTT)3GGG G-quadruplex in complex with N-methyl mesoporphyrin IX
Source: PLoS One. 2020 Nov 18;15(11):e0241513. doi: 10.1371/journal.pone.0241513 (PMC7673559; doi:10.1371/journal.pone.0241513)
Supplement: S1 Fig — (A) TDS and (B-D) CD scans for DNA alone at ~4 μM and with 2 eq. of NMM in 5K buffer at 20 °C. (DOCX) [file pone.0241513.s010.docx]

**S1 Figure.** **CD and TDS signature of T1-NMM, T7-NMM, and T8-NMM.** (**A**) TDS and (**B-D**) CD scans for DNA alone at ~4 µM and with 2 eq. of NMM in 5K buffer at 20 °C.
