# Supplementary material for: Biophysical and X-ray structural studies of the (GGGTT)3GGG G-quadruplex in complex with N-methyl mesoporphyrin IX
Source: PLoS One. 2020 Nov 18;15(11):e0241513. doi: 10.1371/journal.pone.0241513 (PMC7673559; doi:10.1371/journal.pone.0241513)
Supplement: S2 Fig — (A) Representative UV-vis titration of 4.3 μM NMM with 163.5 μM T7 to final [T7]/[NMM] of 2.1. (B) Tit of titration data (solid squares) to the 1:1 binding model with floating concentration of NMM. The 95% confidence interval is shown as dashed lines. (DOCX) [file pone.0241513.s011.docx]

**S2 Figure. Determination of *K_a_* for the T7-NMM complex via UV-vis titration**. (**A**) Representative UV-vis titration of 4.3 µM NMM with 163.5 µM T7 to final [T7]/[NMM] of 2.1. (**B**) Tit of titration data (solid squares) to the 1:1 binding model with floating concentration of NMM. The 95% confidence interval is shown as dashed lines.
