# Supplementary material for: Biophysical and X-ray structural studies of the (GGGTT)3GGG G-quadruplex in complex with N-methyl mesoporphyrin IX
Source: PLoS One. 2020 Nov 18;15(11):e0241513. doi: 10.1371/journal.pone.0241513 (PMC7673559; doi:10.1371/journal.pone.0241513)
Supplement: S3 Fig — T1 data (yellow) was collected with 2–5 eq. of DNA. Fig adapted from (12). (DOCX) [file pone.0241513.s012.docx]

**S3 Figure.** **Fluorescence enhancement data for NMM in the presence of 10 eq. of the indicated DNA sequences**. T1 data (yellow) was collected with 2-5 eq. of DNA. Figure adapted from [2].

Buffers:

(TB): 50 mM Tris-borate pH 8.3, 10 mM KCl, 1 mM MgCl_2_

(100Li): 10 mM lithium cacodylate pH 7.2, 100 mM LiCl

(5K 5.8): 10 mM lithium cacodylate pH 5.8, 5 mM KCl, 95 mM LiCl

(50Na): 10 mM lithium cacodylate pH 7.2, 50 mM NaCl, 50 mM LiCl.

**

**
