# Supplementary material for: Biophysical and X-ray structural studies of the (GGGTT)3GGG G-quadruplex in complex with N-methyl mesoporphyrin IX
Source: PLoS One. 2020 Nov 18;15(11):e0241513. doi: 10.1371/journal.pone.0241513 (PMC7673559; doi:10.1371/journal.pone.0241513)
Supplement: S4 Fig — Experiments were conducted with ~4.5 μM T1 in 10 mM lithium cacodylate pH 7.2 in the presence of 5–100 mM KCl. (A) CD signatures, (B) CD melting curves, (C) Tm, (D) ΔH, and (E) thermodynamic parameters determined from melting curves. (DOCX) [file pone.0241513.s013.docx]

**S4 Figure.** **Effect of K^+^ concentration on fold and stability of T1**. Experiments were conducted with ~4.5 μM T1 in 10 mM lithium cacodylate pH 7.2 in the presence of 5 - 100 mM KCl. (**A**) CD signatures, (**B**) CD melting curves, (**C**) *T*_m_, (**D**) ΔH, and (**E**) thermodynamic parameters determined from melting curves.

**E**

| [K^+^], mM | *T_m_*, °C | ΔH, kJ/mol | ΔH, kcal/mol | Hysteresis, °C |
| --- | --- | --- | --- | --- |
| 5 | 56.2 ± 0.4 | 197 ± 5 | 47 ± 1 | 3.3 |
| 20 | **65.5 ± 0.9** | **250 ± 10** | **60. ± 3** | **2.9** |
| 30* | 68.3 ± 0.3 | 266 ± 4 | 64 ± 1 | 2.7 |
| 50 | 72 ± 1 | 302 ± 4 | 72 ± 1 | 2.1 |
| 80 | 76.2 ± 1.4 | 334 ± 8 | 80. ± 2 | 1.9 |
| 100* | 76.7 ± 0.3 | 352 ± 6 | 84 ± 1 | 1.2 |

**these numbers were measured once, and the errors come from the instrument uncertainty or data fitting.*
