# Supplementary material for: Biophysical and X-ray structural studies of the (GGGTT)3GGG G-quadruplex in complex with N-methyl mesoporphyrin IX
Source: PLoS One. 2020 Nov 18;15(11):e0241513. doi: 10.1371/journal.pone.0241513 (PMC7673559; doi:10.1371/journal.pone.0241513)
Supplement: S5 Fig — The α, β, γ, δ, ε, and ξ angles characterize the DNA backbone while the χ angle indicates syn vs. anti nucleotide conformation. Each individual angle is shown as a dot. (DOCX) [file pone.0241513.s014.docx]

**S5 Figure. Torsional angle wheel for the (A) T1-NMM and (B) T7-NMM structures**. The α, β, γ, δ, ε, and ξ angles characterize the DNA backbone while the χ angle indicates *syn* vs. *anti* nucleotide conformation. Each individual angle is shown as a dot.

**
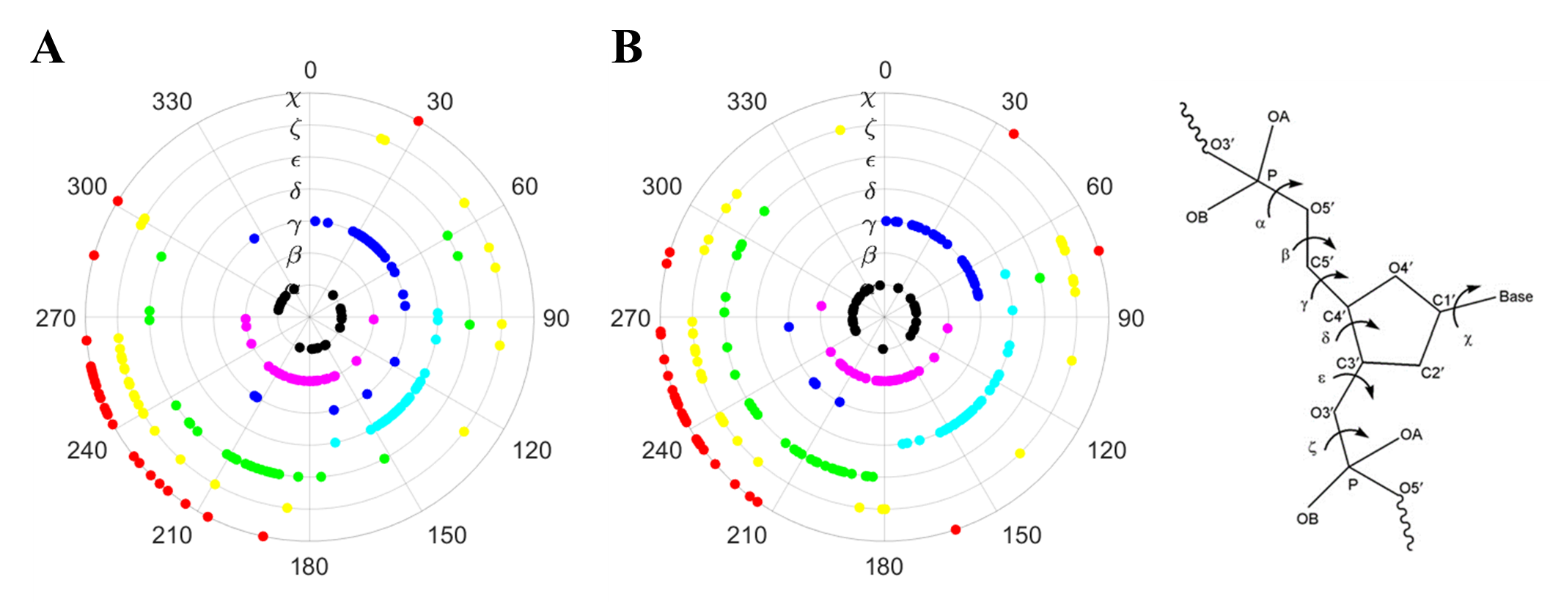
**
