# Supplementary material for: Biophysical and X-ray structural studies of the (GGGTT)3GGG G-quadruplex in complex with N-methyl mesoporphyrin IX
Source: PLoS One. 2020 Nov 18;15(11):e0241513. doi: 10.1371/journal.pone.0241513 (PMC7673559; doi:10.1371/journal.pone.0241513)
Supplement: S6 Fig — (A) The T1-NMM asymmetric unit. (B) Intermolecular interactions among T1 and NMM molecules. As observed in the T7-NMM structure, NMM stacks onto the 3’ G-quartet of one GQ and interacts with a thymine from another GQ (dashed box). Chain A of T1 is colored in teal, chain B is blue, the sugar-phosphate backbone is yellow, and NMM is magenta. Potassium ions are depicted as spheres. (DOCX) [file pone.0241513.s015.docx]

**S6 Figure.** **Intermolecular interactions in the T1-NMM crystal structure**. (**A**) The T1-NMM asymmetric unit. (**B**) Intermolecular interactions among T1 and NMM molecules. As observed in the T7-NMM structure, NMM stacks onto the 3’ G-quartet of one GQ and interacts with a thymine from another GQ (dashed box). Chain A of T1 is colored in teal, chain B is blue, the sugar-phosphate backbone is yellow, and NMM is magenta. Potassium ions are depicted as spheres.
