# Supplementary material for: Biophysical and X-ray structural studies of the (GGGTT)3GGG G-quadruplex in complex with N-methyl mesoporphyrin IX
Source: PLoS One. 2020 Nov 18;15(11):e0241513. doi: 10.1371/journal.pone.0241513 (PMC7673559; doi:10.1371/journal.pone.0241513)
Supplement: S7 Fig — Crystallization samples (cryst) contained 0.65 mM DNA and 0.65 mM NMM (1:1 DNA:NMM) in 20K buffer. Biophysical samples (biophys) contained 5 μM DNA and 10 μM NMM (1: DNA:NMM) if applicable in 5K buffer. (A-B) CD scans at 25 °C in 0.11 mm cuvettes (for cryst) or 10 mm cuvettes (for biophys). (C) Fifteen percent PAGE. Cryst samples were diluted to 50 μM immediately before loading while biophys samples were prepared at 50 μM. The gel was run with 5 mM KCl at 150 V for 150 min at room temperature and visualized using Stains-All. (DOCX) [file pone.0241513.s016.docx]

**S7 Figure.** **Comparison of crystallization and biophysical samples of T1- and T7-NMM**. Crystallization samples (cryst) contained 0.65 mM DNA and 0.65 mM NMM (1:1 DNA:NMM) in 20K buffer. Biophysical samples (biophys) contained 5 μM DNA and 10 μM NMM (1: DNA:NMM) if applicable in 5K buffer. (**A-B**) CD scans at 25 °C in 0.11 mm cuvettes (for cryst) or 10 mm cuvettes (for biophys). (**C**) Fifteen percent PAGE. Cryst samples were diluted to 50 μM immediately before loading while biophys samples were prepared at 50 μM. The gel was run with 5 mM KCl at 150 V for 150 min at room temperature and visualized using Stains-All
