# Supplementary material for: Biophysical and X-ray structural studies of the (GGGTT)3GGG G-quadruplex in complex with N-methyl mesoporphyrin IX
Source: PLoS One. 2020 Nov 18;15(11):e0241513. doi: 10.1371/journal.pone.0241513 (PMC7673559; doi:10.1371/journal.pone.0241513)
Supplement: S1 Raw images — (PDF) [file pone.0241513.s017.pdf]

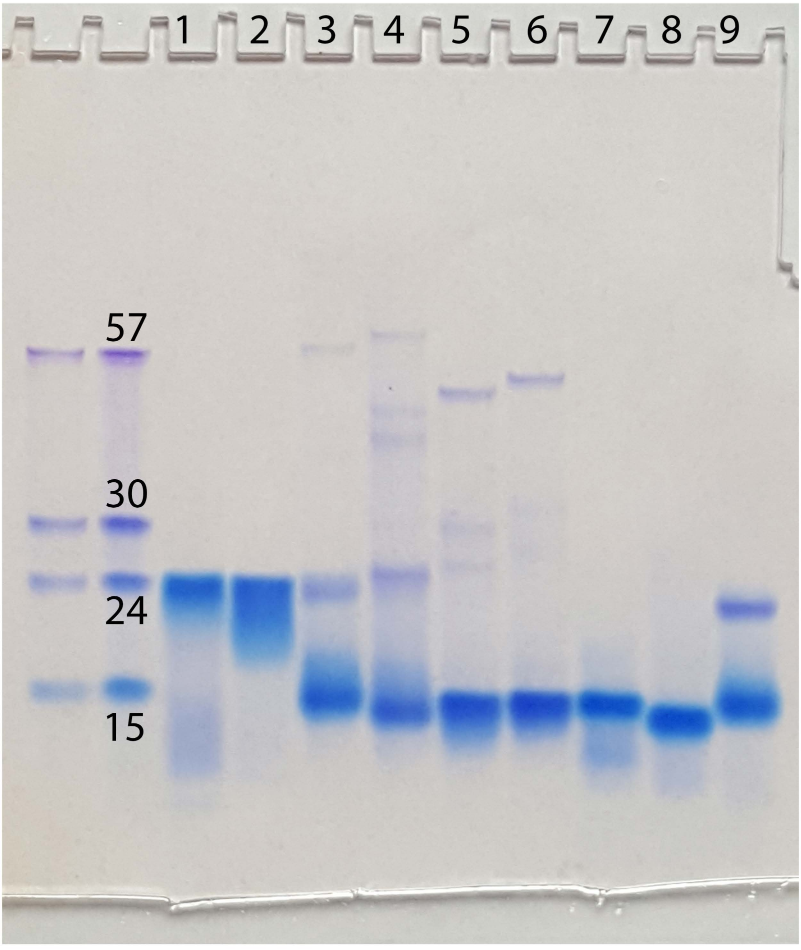
1 2 3 4 5 6 7 8 9
57
30
24
15

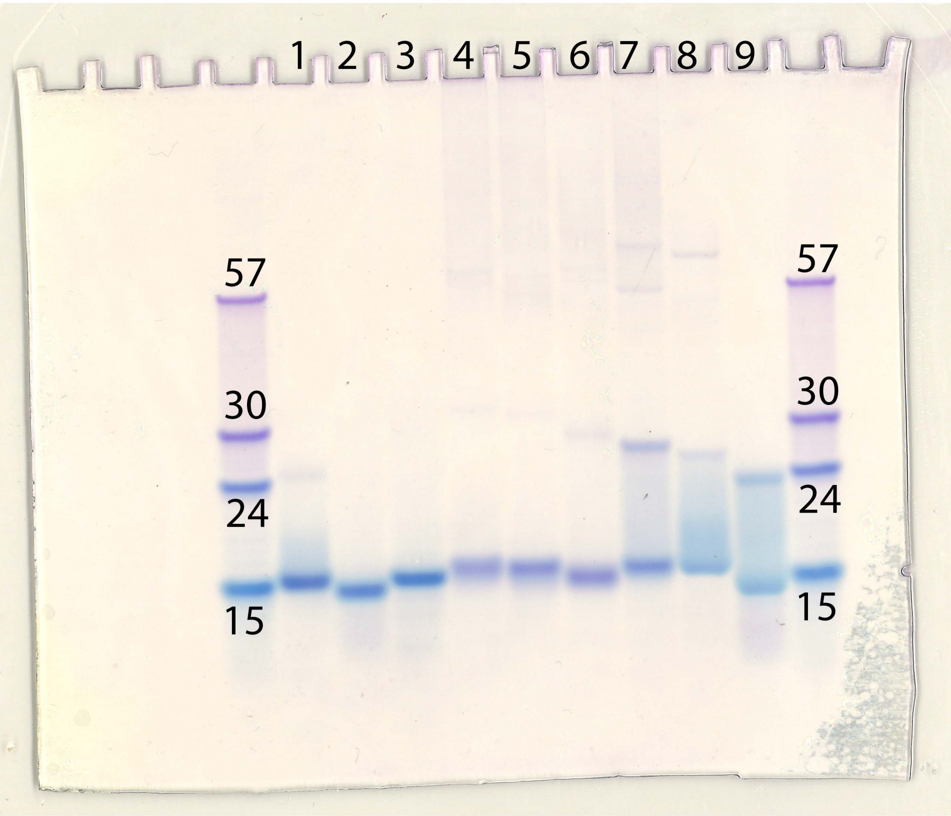
1 2 3 4 5 6 7 8 9
57
30
24
15
57
30
24
15

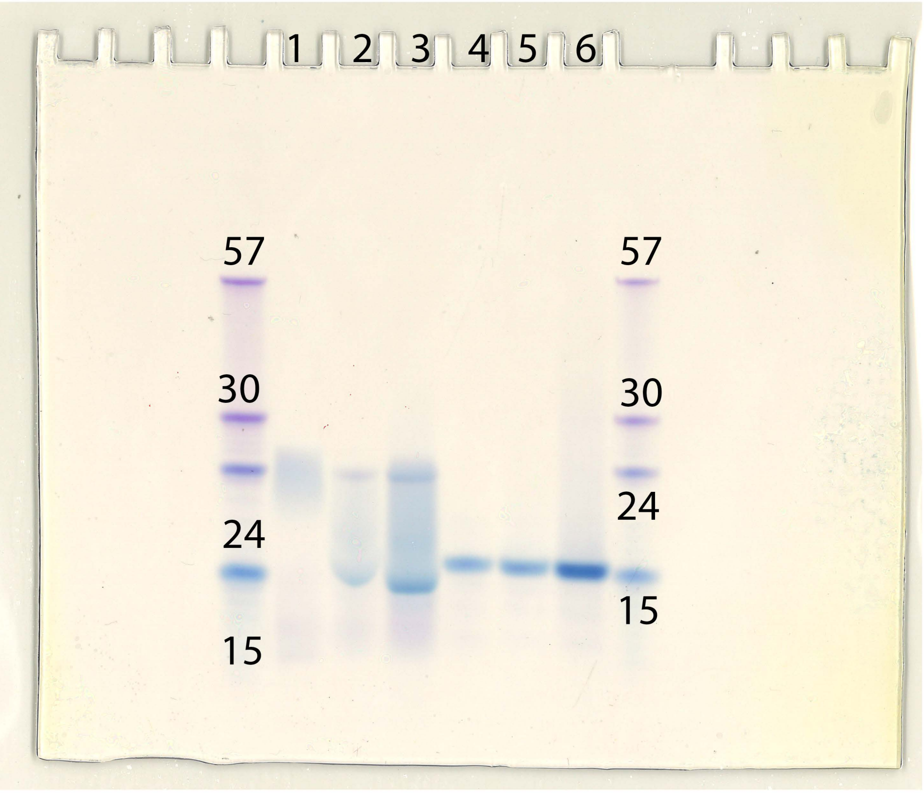
1 2 3 4 5 6
57
30
24
15
57
30
24
15

(Top) Twenty percent native PAGE gels for DNA alone. Lanes 1-9 correspond to T1-T9 constructs.

(Middle) Twenty percent native PAGE gels for DNA alone. Lanes 1-9 correspond to T1-T9 constructs annealed with 2 eq. of NMM.

(Bottom) Fifteen percent PAGE gel.

1 – T1 (biophys)

2 – T1-2eq NMM (biophys)

3 – T1-1eq NMM (cryst)

4 – T7 (biophys)

5 – T7-2eq NMM (biophys)

6 – T7-1eq NMM (cryst)

Crystallization samples (cryst) contained 0.65 mM DNA and 0.65 mM NMM (1:1 DNA:NMM) in 20K buffer. Biophysical samples (biophys) contained 5 μM DNA and 10 μM NMM (1: DNA:NMM) if applicable in 5K buffer. Cryst samples were diluted to 50 μM immediately before loading while biophys samples were prepared at 50 μM.

All gels were run with 5 mM KCl at 150 V for 150 min at room temperature and visualized using Stains-All
