# Supplementary material for: Biophysical and X-ray structural studies of the (GGGTT)3GGG G-quadruplex in complex with N-methyl mesoporphyrin IX
Source: PLoS One. 2020 Nov 18;15(11):e0241513. doi: 10.1371/journal.pone.0241513 (PMC7673559; doi:10.1371/journal.pone.0241513)
Supplement: Attachment — Submitted filename: Response to Reviewers.docx [file pone.0241513.s018.docx]

**Response to Reviewers**

We thank both Reviewers for their positive feedback and useful suggestions. Comments from the referees appear in black and our explanations are in blue; text included in the revised manuscript is in red.

**Table 1: the authors should add the indications of the 5’ and 3’ ends**

Added 5′ → 3′ to the header row of Table 1.

**p.4-5: the authors should better stress the relevance of the studied constructs in human**

We have restructured and expanded the relevant paragraph to stress the relevance of the T1 construct in human:

“We used BLAST (13) to align T1 with a number of genomes. The T1 sequence occurs 11 times in the human genome in seven distinct regions across six chromosomes including within the zinc finger protein 292 gene; on the complementary strand of the plexin A4 gene; within the C-type lectin domain family 2 member A gene; carboxypeptidase M gene, *fas* binding factor 1 gene; and LOC107984696 non-coding RNA (**Table S1**). T1 may also be involved in regulating *β*-secretase 2 (BACE2), a gene implicated in Alzheimer’s disease (17). In addition, the T1 sequence is found at position 168,273 in the *Tetrahymena thermophila* genome in the telomeric region. However, it does not represent the common telomeric motif, (GGGGTT)_n_ (crystallographic studies on this consensus motif are underway in our laboratory). Recently, a protein that binds parallel telomeric GQs (*K_d_* = 11.5 μM) was discovered in T. thermophila (16), thereby providing compelling evidence for the biological relevance of such GQ structures. Finally, T1 sequence is found in the bacteria *Neisseria meningitidis*, *Neisseria gonorrhoeae*, the bird pathogen *Escherichia coli* strain APEC O78, and repetitively in *Paenibacillus* (18,19).”

We have done the same in the Conclusion part:

“The T1 and T7 sequences are found in the human genome (including a gene implicated in Alzheimer’s disease (14)); in the *T. thermophila* telomere (but do not represent the telomeric repeat); and in the genomes of at least 34 bacteria (including pathogenic species) (16,17).”

**p.5-7, p.36-37: it seems that the sequence of the reference numbering is incorrect. Please check**

Checked and corrected

**p.5 l.95: the search in the pdb database using the key words and refinements parameters reported, provided a higher numbers of entries**

We performed the search with the key words “"G-quadruplex Ligand". Bulk of the entries contained K^+^, Na^+^, and Mg^2+^ as ligands, and those were removed. Out of the other entries, only unique structures were counted. For example, there are two structures of human telomeric DNA with N-methylmesoporphyrin IX from our lab but we counted them as one entry only.

We have added the following clarifying sentence:

These numbers were obtained by searching PDB for "G-quadruplex Ligand", refining the search parameters by "DNA" and either "X-ray Diffraction" or "Solution NMR", and manually examining all entries keeping those that are unique.

**p.16 Table 2: the authors should explain why the ligand (NMM) has different atom numbers in the two structures**

All atoms in the T7-NMM structure were modeled at full occupancy, whereas one NMM molecule and one potassium ion in the T1-NMM structure were modeled in two different positions each at 0.5 occupancy. In **Table 2** every atom in NMM was counted, including those modeled in two positions, leading to doubling the numbers. In addition, “ligand” in **Table 2** refers to NMM, potassium ions and sodium formate molecules. Below is a detailed breakdown of the identities of the atoms counted in the Table 2:

|  | **T1-NMM** | **T7-NMM** |
| --- | --- | --- |
| **NMM bound to chain A**  **(built without propionate groups)** | 35 | 35 |
| **NMM bound to chain B**  **(built without propionate groups)** | 35+35  *(NMM in chain B was modeled in two different orientations each at 0.5 occupancy)* | 35 |
| **Total NMM atoms** | 105 | 70 |
| **Potassium ions** | 6  *(There are five K^+^ with the one at the dimer interface modeled at 0.5 occupancy)* | 5 |
| **Sodium formate** | 4 | 0 |
| **Total ligand atoms** | **115** | **75** |

Based on the comment from the reviewer, we have revised **Table 2** to clearly indicate that both structures have two NMM molecules and five potassium ions:

**Table 2. Crystallographic statistics for the T1-NMM and T7-NMM complexes.**

| Number of atoms | 822* | 862 |
| --- | --- | --- |
| DNA | 707 | 783 |
| NMM | 70 | 70 |
| water | 0 | 4 |
| Potassium | 5 | 5 |
| Sodium formate | 4 | 0 |

**NMM molecule bound to chain B was modeled in two different orientation each at 0.5 occupancy; one potassium ion rests on the special position at the dimer interface and was modeled at 0.5 occupancy in each monomer. The total number of atoms, 822, in T1-NMM results from counting every atom of NMM in each of the two position as well as an additional potassium (five total, counted as six due to split occupancy of one K^+^).*

**p.27 l.535-537: please rephrase**

(Preceding sentence: “In addition, the 5’ and 3’ thymine overhangs in T7 (not present in T1) are highly disordered, with a B-factor of 151.63 Å^2^.”)

“The flexibility of these terminal thymines, along with the potential of these overhangs to destabilize the dimer observed in solution for T1 but not T7, may explain the destabilization of T7 with respect to T1 by -5.8 °C (**Table 1**).”

We have rephrased the whole paragraph in the following way:

T1-NMM and T7-NMM have high overall B-factors, 115.44 and 97.15 Å^2^, respectively (**Table S5**). Main contributors are flexible disordered loops whose B-factors are 148.13 Å^2^ for T1-NMM and 128.85 Å^2^ for T7-NMM. T7 also contains 5’ and 3’ thymine overhangs with an average B-factor of 151.63 Å^2^. The flexibility of these terminal thymines may explain the destabilization of T7 with respect to T1 by -5.8 °C (**Table 1**).

**p.28 l.578-580: please specify the distance**

“NMM binds to the 3’ G-quartet at a distance of 3.6 Å in both the T1- and T7-NMM structures (**Table S6**), as well as in the Tel22-NMM structure (28). This distance is slightly longer than the optimal π-π stacking distance of 3.4 Å.”

We have specified the distance in the experimental section:

Distances between adjacent G-quartets and between the outermost G-quartet and NMM were calculated using the centroid of each G-quartet and of NMM (using the atomic coordinates of the 24 atoms comprising the porphyrin ring).

We have also added the following sentence to the Result section for clarification:

This distance was measured between the centroids of NMM and the 3’ G-quartet.

**The quality of Figures 6 and 7 is poor. Please provide pictures of higher quality**

We have provided higher quality figures.
